# Primary adrenal insufficiency in adult population: a Portuguese Multicentre Study by the Adrenal Tumours Study Group

**DOI:** 10.1530/EC-17-0295

**Published:** 2017-10-31

**Authors:** Lia Ferreira, João Silva, Susana Garrido, Carlos Bello, Diana Oliveira, Hélder Simões, Isabel Paiva, Joana Guimarães, Marta Ferreira, Teresa Pereira, Rita Bettencourt-Silva, Ana Filipa Martins, Tiago Silva, Vera Fernandes, Maria Lopes Pereira, Adrenal Tumors Study Group of the Portuguese Society of Endocrinology

**Affiliations:** 1 Department of EndocrinologyCentro Hospitalar do Porto, Porto, Portugal; 2 Department of EndocrinologyHospital das Forças Armadas, Lisboa, Portugal; 3 Department of EndocrinologyCentro Hospitalar Tâmega e Sousa, Porto, Portugal; 4 Department of EndocrinologyCentro Hospitalar Lisboa Ocidental, Lisboa, Portugal; 5 Department of EndocrinologyCentro Hospitalar e Universitário de Coimbra, Coimbra, Portugal; 6 Department of EndocrinologyInstituto Português de Oncologia de Lisboa Francisco Gentil, Lisboa, Portugal; 7 Department of EndocrinologyCentro Hospitalar do Baixo Vouga, Aveiro, Portugal; 8 Department of EndocrinologyCentro Hospitalar de Leiria, Leiria, Portugal; 9 Department of EndocrinologyCentro Hospitalar de São João, Porto, Portugal; 10 Department of EndocrinologyCentro Hospitalar Lisboa Norte, Lisboa, Portugal; 11 Department of EndocrinologyHospital Garcia da Orta, Lisboa, Portugal; 12 Department of EndocrinologyHospital de Braga, Braga, Portugal

**Keywords:** primary adrenal insufficiency, Addison disease, glucocorticoid replacement, mineralocorticoid replacement, Portugal

## Abstract

**Introduction:**

Primary adrenal insufficiency (PAI) is a rare but severe and potentially life-threatening condition. No previous studies have characterized Portuguese patients with PAI.

**Aims:**

To characterize the clinical presentation, diagnostic workup, treatment and follow‐up of Portuguese patients with confirmed PAI.

**Methods:**

This multicentre retrospective study examined PAI patients in 12 Portuguese hospitals.

**Results:**

We investigated 278 patients with PAI (55.8% were females), with a mean age of 33.6 ± 19.3 years at diagnosis. The most frequent presenting clinical features were asthenia (60.1%), mucocutaneous hyperpigmentation (55.0%) and weight loss (43.2%); 29.1% of the patients presented with adrenal crisis. Diagnosis was established by high plasma ACTH and low serum cortisol in most patients (43.9%). The most common aetiology of PAI was autoimmune adrenalitis (61.0%). There were 38 idiopathic cases. Autoimmune comorbidities were found in 70% of the patients, the most frequent being autoimmune thyroiditis (60.7%) and type 1 diabetes mellitus (17.3%). Seventy-nine percent were treated with hydrocortisone (mean dose 26.3 ± 8.3 mg/day) mostly in three (57.5%) or two (37.4%) daily doses. The remaining patients were treated with prednisolone (10.1%), dexamethasone (6.2%) and methylprednisolone (0.7%); 66.2% were also on fludrocortisone (median dose of 100 µg/day). Since diagnosis, 33.5% of patients were hospitalized for disease decompensation. In the last appointment, 17.2% of patients had complaints (7.6% asthenia and 6.5% depression) and 9.7% had electrolyte disturbances.

**Conclusion:**

This is the first multicentre Portuguese study regarding PAI. The results emphasize the need for standardization in diagnostic tests and etiological investigation and provide a framework for improving treatment.

## Introduction

Addison disease (AD) or primary adrenal insufficiency (PAI) is a life-threatening disease that results from bilateral destruction or dysfunction of the adrenal cortex (1). PAI is a rare disease with a reported prevalence of approximately 100 per million inhabitants in European countries. The incidence has been estimated at 4–6 cases per million per year but seems to be increasing (2, 3, 4, 5, 6, 7, 8).

Tuberculosis has historically been the predominant cause of AD (9, 10, 11, 12), but autoimmune adrenalitis is now the most prevalent aetiology (80–90%) in industrialized countries (1, 4, 5, 7, 13). Other causes include infectious, genetic, metastatic, haemorrhagic and infiltrative disorders, as well as surgery or drugs, but these cases are rare in adult patients.

Autoimmune adrenalitis is frequently associated with other organ-specific autoimmune disorders and type 1 or type 2 autoimmune polyendocrine syndrome (APS-1 and APS-2). APS-1 is a very rare monogenic disorder caused by mutations in the autoimmune regulator (*AIRE*) gene and is characterized by the presence of two of the three main components: AD, chronic mucocutaneous candidiasis and hypoparathyroidism. The more common APS-2 is characterized by two or more concurrent autoimmune endocrinopathies, such as AD, autoimmune hypo- or hyperthyroidism and type 1 diabetes mellitus (DM) (14). Isolated PAI and APS-2 share the same pattern of complex inheritance (7).

To date, no data have been available about the epidemiology, clinical features and management of Portuguese patients with PAI. The main objective of this study is to characterize the clinical presentation, diagnostic workup, treatment and follow‐up of such patients. Other objectives of this study are to explore the treatment modalities in this group and identify the most prevalent concomitant diseases.

## Methods

This observational, multicentre, retrospective study investigated patients older than 18 years with a diagnosis of PAI followed by endocrinology in 12 national hospitals (four in Northern Portugal, three in Central Portugal, and five in Southern Portugal). Hospitals in the Adrenal Tumours Study Group (GET-SR) of the Portuguese Society of Endocrinology were invited to participate in the study. Patient selection and data collection occurred between September and November 2016. All patient clinical data were anonymized and analysed by an independent reviewer. Consent has been obtained from each patient after full explanation of the purpose and nature of all procedures used. Ethical approval was granted by the Ethics Committee of Centro Hospitalar de São João.

The diagnosis of PAI was verified by review of medical records by 15 endocrinologists and endocrinology residents from the 12 Portuguese centres. AD was diagnosed based on one of the following criteria: (1) a plasma adrenocorticotropic hormone (ACTH) concentration exceeding 66 pmol/L in combination with a low serum cortisol (<5 µg/dL); (2) an abnormal standard dose ACTH stimulation test (peak plasma cortisol <18 µg/dL); (3) characteristic clinical signs and symptoms such as hyperpigmentation, salt craving, typical electrolyte disturbances and chronic treatment with glucocorticoids and fludrocortisone in patients who were diagnosed decades ago. The diagnosis of autoimmune AD was based on positive autoantibodies toward 21-hydroxylase or associated autoimmune diseases.

Cases with no known cause were classified as idiopathic. Patients were excluded if their adrenal insufficiency was deemed to be secondary to adrenalectomy, pituitary surgery or prolonged use of glucocorticoids. Patients who died since diagnosis were also excluded.

We studied patient demographic and clinical data, particularly regarding diagnosis, aetiology, concomitant autoimmune disorders, family history and replacement regimens, such as the type, daily dosage and dose frequency of glucocorticoids, as well as the use of mineralocorticoids. We assessed the occurrence of hospitalization due to adrenal crisis through the review of all hospital medical records since the diagnosis. Adrenal crisis (AC) was defined as an acute impairment of general health requiring hospital admission and administration of intravenous saline and glucocorticoids in patients with AD. Furthermore, symptoms and electrolyte disturbances at the last appointment were evaluated.

A comparative analysis was performed to investigate the type of glucocorticoid replacement and aetiology, mean daily hydrocortisone equivalent dose, mean daily fludrocortisone dose, occurrence of AC and the presence of symptoms or electrolyte disturbances. Data were initially collected in an Excel 2011 database, and statistical analysis was performed with SPSS Statistics, V.22. Data are presented as proportions, means (s.d.) or medians (range) for variables that did not conform to a normal distribution. The crude incidence rates of hospitalization due to AC were estimated as the number of hospitalization cases divided by the total follow-up time and were reported as number of cases per 100 patient-years. For independent samples, two-way comparisons for proportions were performed using a chi-square test (*χ*²) for categorical variables and a *t*-test or Mann–Whitney *U* test for continuous variables. Statistical significance was taken as two-tailed at the level of 0.05. All phases of preparation for the study were in line with the ethical and deontological principles regarding data collection and statistical analysis.

## Results

The study included 278 adult patients diagnosed with AD between 1950 and 2016, of which 56% were female. The mean age at diagnosis was 33.6 ± 19.3 years.

### Clinical presentation

At the time of the diagnosis, the most frequently reported symptoms and signs were asthenia (60.1%), mucocutaneous hyperpigmentation (55.0%), weight loss (43.2%), hypotension (42.8%) and hypoglycaemia (8.6%). Hyponatraemia was documented in 36.3% of cases, while hyperkalaemia occurred in 25.9%. AC was diagnosed in 29.1% of the patients. The median lag time between onset of symptoms and confirmed diagnosis was 3 (0–36) months.

### Diagnosis workup

The diagnoses were established by high ACTH and low serum cortisol in 122 (43.9%) patients; by an abnormal standard dose ACTH stimulation test (peak plasma cortisol <18 µg/dL) in 39 (14.0%) patients and by characteristic clinical symptoms/signs and chronic treatment with glucocorticoids and fludrocortisone in 48 (17.3%) patients who were diagnosed decades ago. These data were missing for 69 (24.8%) patients.

**Figure 1:**
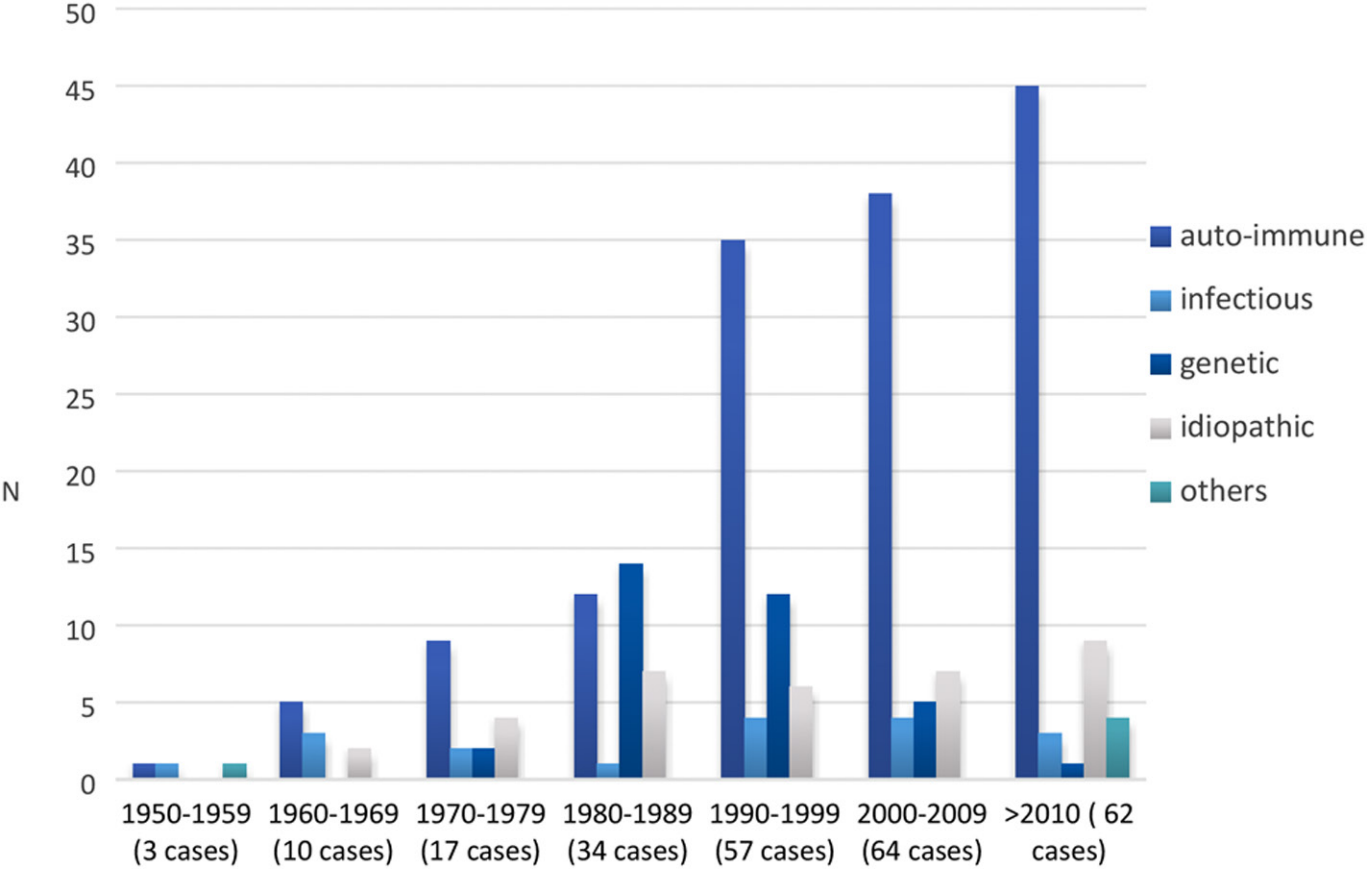
Frequency of AD cases according to date of diagnosis and aetiology.

Data regarding etiological investigation were available in 246 patients. The most common causes were autoimmune adrenalitis (61.0%), genetic-related AD (14.2%) and infectious adrenalitis (7.3%). Thirty-eight cases (15.4%) were considered idiopathic. We also documented three cases secondary to bilateral adrenal haemorrhage and two cases of adrenal metastatic infiltration. Figure 1 summarizes the frequency of different forms of AD diagnosed from 1950 to 2016.

### Patients with autoimmune AD

Among 150 patients with autoimmune AD, 65.3% were female and the mean age at diagnosis was 34.9 ± 15.9 years. The mean age of diagnosis was higher for females, 37.9 ± 15.3 years than for males, 29.2 ± 15.5 years (*P* < 0.001).

Ninety-three percent of the patients had autoantibodies directed against 21-hydroxylase. Computed tomography (CT) scans of the adrenal glands were performed in 50 patients, among which adrenal morphology was described as normal in 37 cases, while 13 patients presented reduced size or volume.

### Associated endocrine and autoimmune disorders

**Figure 2:**
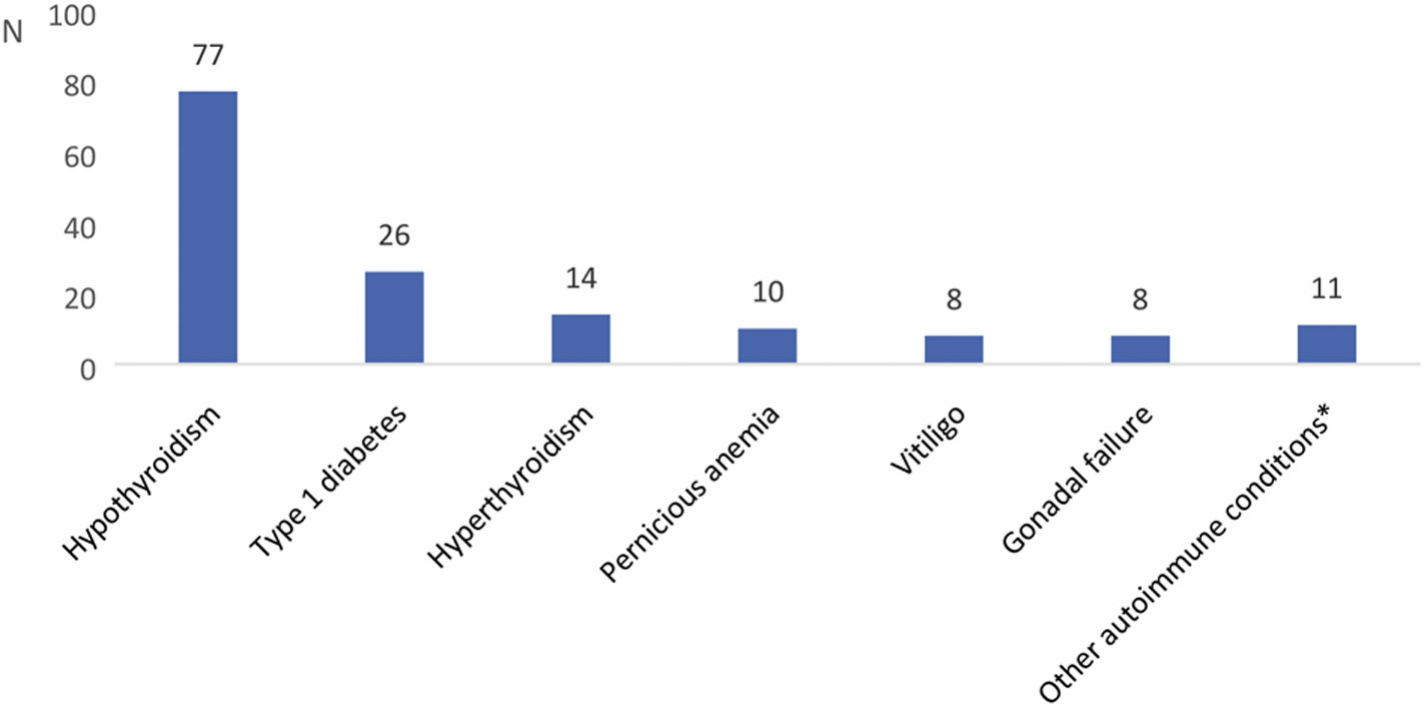
Frequency of concomitant endocrine and autoimmune diseases in patients with autoimmune AD. *other: chronic mucocutaneous candidiasis, hypoparathyroidism, alopecia.

Isolated autoimmune AD occurred in 30% of the patients, whereas 70% were diagnosed with one or more associated autoimmune endocrinopathies. The most common of these were autoimmune thyroiditis (60.7%), type 1 diabetes mellitus (17.3%) and pernicious anaemia (6.7%), with 103 (68.7%) of the patients meeting the criteria for APS-2. We also found two patients with associated chronic mucocutaneous candidiasis and meeting the criteria for APS-1. Other recorded comorbidities are presented in Fig. 2.

### Patients with genetic forms of AD

Congenital adrenal hyperplasia (CAH) resulting from 21-hydroxylase deficiency occurred in 27 patients (55.6% female), with a median age at onset of 3 (range, 0–30) years. The diagnosis of CAH was based on the molecular analysis of CYP21A2 gene. Five patients had X-linked adrenoleukodystrophy (ALD), with a median age at diagnosis of 25 (range, 11–28) years. Three patients had X-linked congenital adrenal hypoplasia with a mutation of DAX1, who were all diagnosed within the first year of life.

### Patients with post-infectious AD

Out of the 18 cases (50% female) of infectious adrenalitis, 17 resulted from tuberculosis and one resulted from histoplasmosis. The median age at diagnosis was 50 (range, 18–69) years. Ten patients had records of radiological studies, of which seven showed an increase in adrenal volume and three had adrenal glands with normal morphology.

### Idiopathic AD

In the 38 cases considered idiopathic, the median age at diagnosis was 41 (range, 5–78) years. Only two of these patients had records of anti-adrenal antibodies (negative), and 18 patients had radiological imaging of the adrenal glands (the adrenals were reduced in size/volume with calcifications in five patients and normal in 13 patients).

### Treatment and follow-up

All patients received glucocorticoid replacement therapy, 79.1%t were treated with hydrocortisone, 10.1% with prednisolone, 6.1% with dexamethasone and 0.7% methylprednisolone (missing data = 11). The mean daily dose for patients with hydrocortisone treatment was 26.3 ± 8.3 mg/day (missing data = 1), which was divided into one (3.5%), two (37.4%), three (57.5%) or four (0.5%) daily doses (missing data = 3). Other glucocorticoid replacement doses were recalculated as the hydrocortisone equivalent dose (Table 1). Patients receiving hydrocortisone had a significantly higher mean daily hydrocortisone equivalent dose than patients receiving prednisolone (mean difference of 4.7 ± 1.6 mg/day; *P* < 0.05) and dexamethasone (mean difference of 15.2 ± 2.0 mg/day; *P* < 0.001). Replacement with dexamethasone was significantly more frequent among patients with CAH (82.4% vs 17.6%; *P* < 0.001). There were no differences between the other types of glucocorticoid replacement and aetiology.

**Table 1:** Glucocorticoid and mineralocorticoid replacement.

**Glucocorticoid therapy** (*N* = 267)	**%**	**Mean daily hydrocortisone equivalent dose** (mg)	**Doses/day** (%)				**Mineralocorticoid therapy** (%)	**Median daily dose of fludrocortisone** (µg)
			1	2	3	4		
Hydrocortisone	79.1	26.3 ± 8.3	3.7	37.4	57.5	0.5	68.9	100 (range 25–200)
Prednisolone	10.1	21.6 ± 7.4	100	–	–	–	88.9	100 (range 50–200)
Dexamethasone	6.1	11.1 ± 7.4	100	–	–	–	52.9	100 (range 50–150)
Methylprednisolone	0.7	20	100	–	–	–	0	–
Total	100	24.8 ± 8.9	15.8	33.1	45.7	0.7	66.5	100 (range 25–200)

Mineralocorticoid replacement was utilized by 66.5% of the patients, and the median fludrocortisone dose was 100 (range, 25–200) µg/day. The two patients under methylprednisolone replacement did not receive mineralocorticoid. The prevalence of fludrocortisone replacement was higher in patients with prednisolone (88.9%) than in patients with hydrocortisone (68.9%; *P* = 0.021) or dexamethasone (52.9%; *P* = 0.01), but there were no differences regarding daily fludrocortisone dose (*P* = 0.46). The mean hydrocortisone equivalent dose was not significantly different in patients with or without mineralocorticoid replacement (24.0 ± 8.1 mg/day vs 25.1 ± 9.3 mg/day; *P* = 0.35).

Since the diagnosis, 33.5% of patients had been hospitalized due to AC. The overall incidence of AC was 4.36 (95% CI 3.98–4.74) per 100 patient-years. The incidence rate of hospital admission was higher in the group receiving hydrocortisone (4.94, 95% CI 4.51–5.34) than in the group receiving prednisolone (2.78, 95% CI 1.99–3.56) per 100 patient-years (*P* = 0.001). There were no differences between dose of glucocorticoid, mineralocorticoid replacement or aetiology and the incidence of hospitalizations.

At the last appointment, 17.2% of the patients had complaints (missing data = 31) with most common being asthenia (7.6%) and depression (6.5%), while 9.7% presented electrolyte disturbances (missing data = 17). No association was found between complaints and the type of glucocorticoid replacement, mineralocorticoid therapy, the presence of symptoms or electrolyte disturbances.

## Discussion

We reviewed 278 adult patients with AD followed by endocrinology in 12 Portuguese hospitals. To date, this is the only Portuguese cohort of patients with PAI. Several patients may be followed in other centres or by other specialties, so we were not able to estimate the prevalence of this disease in Portugal. However, considering the dimension and the good distribution of patients across the country, we assume that this cohort is representative of the Portuguese population with AD.

Several recent studies have suggested an increasing incidence of the disease in the last decades (2, 3, 4, 8, 15). In our study, it was not possible to determine the incidence of AD over time, however, in agreement with recent data, we found an increasing relative proportion of autoimmune AD cases and a decrease in infectious etiologies over the last decades (1, 4, 13). These findings are consistent with the widespread increase in autoimmune diseases and the decline in the prevalence of tuberculosis in industrialized countries (3). In line with the results from Lauretta and coworkers, the majority of patients in this cohort were female (5). This difference was more evident in patients with autoimmune AD (64.3% vs 35.7%; *P* = 0.017).

In our cohort, only 14% of patients had records of a corticotropin test. Current guidelines by the Endocrine Society suggest that only if a corticotropin test is not feasible should the combination of low plasma cortisol and ACTH >2 times the upper reference limit be used as a preliminary test for suggesting adrenal insufficiency (16). However, in our study, most cases were diagnosed several decades ago when the availability of corticotropin was limited.

The most common aetiology was autoimmune AD, and 68% of this subset of patients had other autoimmune endocrinopathy. The most frequent concomitant diseases were hypothyroidism, DM, hyperthyroidism and pernicious anaemia, which is in concordance with studies from Norway (7), Italy (1), the United Kingdom (UK) (17), Sweden (18) and Poland (19).

We found 35 patients with genetic forms of adrenal insufficiency, and CAH was the most frequent. CAH is the most common cause of adrenal insufficiency in infancy (20). Considering the exclusion of paediatric patients and the possible loss of continuity of endocrine specialist care from the paediatric to adult environments, we assume that the real number of patients with CAH and probably other genetic forms of AD is underestimated in our study.

The frequency of idiopathic cases was somewhat higher than that reported in other studies (13, 21). Only two patients had records of anti-adrenal antibodies, and 18 had radiological studies of the adrenals. Considering the radiological findings in some of the patients defined as idiopathic, these may correspond to a fraction of underdiagnosed cases of autoimmune or tuberculosis AD. Current guidelines recommend that the aetiology of PAI be determined in all patients. According to the patients’ clinical pictures and family histories, etiologies can be screened using 21-hydroxylase antibodies and the 17-hydroxyprogesterone serum level at baseline. If antibodies are negative, a CT scan of the adrenals may reveal evidence of adrenal infiltrative processes or metastases, and male patients should be tested for adrenoleukodystrophy with plasma levels of very-long-chain fatty acids (16, 22). The high number of idiopathic cases in this study likely reflects the incomplete etiological study of some of these patients.

Current treatment recommendations suggest using short-acting glucocorticoids, hydrocortisone (15–25 mg) or cortisone acetate (20–35 mg) in two or three divided oral doses per day to avoid overtreatment and enable a diurnal physiological pattern (22). Our study revealed that Portuguese patients primarily use short-acting glucocorticoids, which are mostly divided into three daily doses. We found similar data from other European countries in the literature (1, 7, 18). The mean daily equivalent hydrocortisone dose found in this study (26.3 mg/day) was slightly higher than the recommended dose (16). Lower doses have been reported in studies in the United Kingdom (24.0 mg/day) and Italy (25.2 mg/day) (17, 21), but the use of higher doses was found in a Norwegian study (32.4 mg/day) (7). Furthermore, we found that patients receiving long-acting glucocorticoids used a significantly lower mean hydrocortisone equivalent dose than patients on conventional hydrocortisone replacement. However, these preparations are associated with a non-physiological plasma cortisol profile and may result in symptoms of over- or under-substitution, despite optimal dose steroid replacement.

Mineralocorticoid replacement therapy with fludrocortisone is recommended in all patients with PAI (16). The mineralocorticoid replacement rate found in this study (66.5%) was somewhat lower than that reported by other studies. In Swedish and UK cohorts, 89% and 100% of patients were on fludrocortisone, respectively (17, 18).

The incidence rate of hospitalizations due to AC found in this population was lower than that reported in Dutch (5.2/100 person-year) and German (6.3/100 patient-years) patients (23, 24). However, the exclusion of deaths from the assessment is likely to lead to an underestimation of the real incidence of AC in this study, as there is a mortality rate associated with AC (25, 26) and moreover some of these events may have occurred outside hospital. The higher incidence of AC found in patients receiving hydrocortisone may be related with the likelihood that patients on short-acting glucocorticoid replacement therapy have periods of relatively profound hypocortisolaemia that may predispose patients to an AC (27). This finding also emphasizes the importance of patient education concerning glucocorticoid adjustments in stressful events and AC prevention strategies.

On the last appointment, 17% of our patients had complaints, and 10% had electrolyte disturbances. Unfortunately, it was not possible to evaluate in this study the morbidity and mortality associated with AD. There is growing evidence that current replacement regimens are associated with a significant reduction in health status and working ability (28, 29). Moreover, premature death and increased mortality have been demonstrated among AD patients (26). These findings are thought to be due to the non-physiological nature of conventional replacement therapy (30). Delayed-release hydrocortisone preparations have been introduced with the aim of better mimicking the endogenous circadian cortisol rhythm, which have been shown to improve quality of life in patients with AD (31). However, these preparations are still not available in Portugal.

One of the strengths of this study is the inclusion of patients from most tertiary centres in the northern, central and southern regions of Portugal. It is also the first national cohort of patients with AD, and it was possible to characterize the clinical presentation, diagnosis, treatment and follow-up of the disease.

The main limitations of this study stem from its retrospective observational nature in which data were collected from medical records by several investigators. We cannot draw any conclusions about the real incidence of the disease in our country, as this cohort just includes adult patients followed on endocrinology department. Furthermore, as aforementioned, the exclusion of deaths probably leads to an underestimation of the disease and incidence of AC.

In conclusion, autoimmune AD is the most frequent aetiology in this population, but there is still a high prevalence of cases considered idiopathic. This study emphasizes the need for standardization in diagnostic tests, etiological investigation and the investigation of associated autoimmune comorbidities. It is also important to advocate the treatment of these patients according to current recommendations and to minimize symptoms associated with over- or under-substitution of glucocorticoids and under replacement of mineralocorticoid.

## Declaration of interest

The authors declare that there is no conflict of interest that could be perceived as prejudicing the impartiality of the research reported.

## Funding

This research did not receive any specific grant from any funding agency in the public, commercial or not-for-profit sector.
